# Where are the Excess Electrons in Subvalent Compounds? The Case of Ag_7_Pt_2_O_7_

**DOI:** 10.1021/acs.inorgchem.3c04409

**Published:** 2024-03-18

**Authors:** Fernando Izquierdo-Ruiz, Miguel Angel Salvadó, Alvaro Lobato, Jose Manuel Recio

**Affiliations:** † MALTA-Consolider Team and Departamento de Química Física, Universidad Complutense de Madrid. E-28040 Madrid, Spain; ‡ MALTA-Consolider Team and Departamento de Química Física y Analt́ica, Universidad de Oviedo. E-33006 Oviedo, Spain

## Abstract

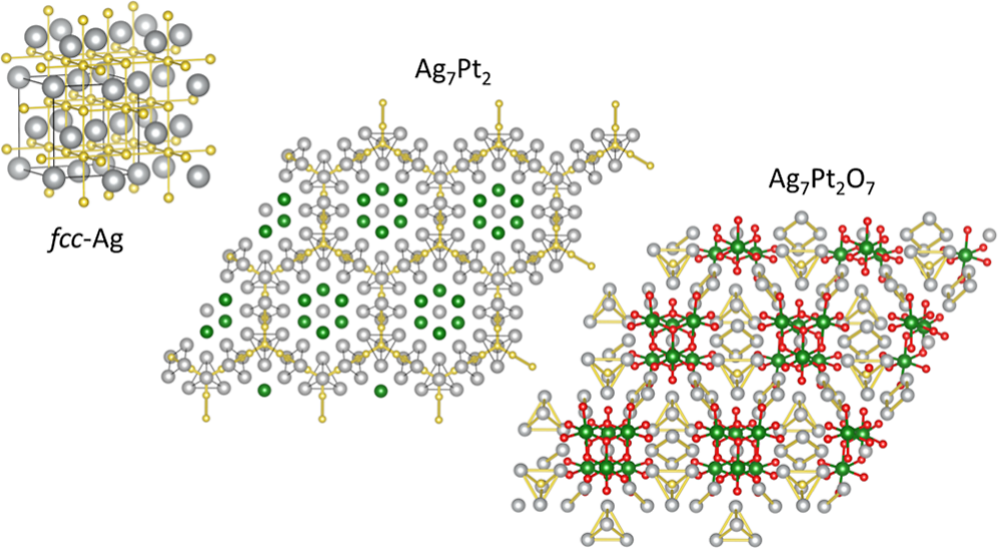

Subvalent compounds raise the question of where those valence electrons not belonging to chemical bonds are. In the limiting case of Ag_7_Pt_2_O_7_, there is just one-electron excess in the chemical formula requiring the presence of Ag atoms with oxidation states below +1, assuming conventional Pt^4+^ and O^2–^ ions. Such a situation challenges the understanding of the semiconducting and diamagnetic behavior observed in this oxide. Previous explanations that localize pairwise the electron excess in tetrahedral Ag_4_ interstices do not suffice in this case, since there are six silver tetrahedral voids and only an excess of nine electrons in the unit cell. Here, we provide an alternative explanation for the subvalent nature of this compound by combining interatomic distances, electron density-based descriptors, and orbital energetic analysis criteria. As a result, Ag atoms that do not participate in their valence electron are revealed. We identify excess electrons located in isolated subvalent silver clusters with electron-deficient multicenter bonds resembling *pieces* of metallic bonding in fcc-Ag and Ag_7_Pt_2_ alloy. Our analysis of the electronic band structure also supports the multicenter bonding picture. This combined approach from the real and reciprocal spaces reconciles existing discrepancies and is key to understanding the *new* chemistry of silver subvalent compounds.

## Abstract 2

The anomalous composition of Ag_7_Pt_2_O_7_ is a result of the preference of the silver valence electrons to keep the electronic circuits (yellow) of the metal rather than to be transferred to the platinum oxide fragment. The electron excess is thus revealed by multicentric bonding present in the Ag-isles of the crystal. The 3D-electronic circuit of the fcc-Ag metal breaks up into a 2D circuit in the Ag_2_Pt_7_ alloy and collapses into isolated clusters in the Ag_7_Pt_2_O_7_ compound.

## Introduction

Subvalent compounds defy traditional chemical bonding rules involving conventional oxidation states since their atoms contribute to the bonding network with fewer valence electrons than expected from their electronic configuration. In the family of silver compounds, the anomalous composition inherent to subvalence also opens the door to new chemistry,^1,2^ where metallophilic interactions^3,4^ change the conventional chemical view due to the participation of closed-shell d^10^ orbitals in spd hybridization.^5−7^ Thanks to the interplay between metallic and ionic bonding, subvalent compounds can be considered “for the design of next-generation multifunctional materials”, with specific applications in the energy storage field.^8,9^ However, a general framework able to unveil structure–property relationships has not yet been established and becomes necessary to advance the understanding of these subvalent materials.

Silver compounds with anomalous composition are currently understood in terms of localized pairwise electrons in tetrahedral Ag_4_^1,2,10,11^ or octahedral Ag_6_^12−15^ voids of the structure. This view provides a consistent picture when it is applied to explain the subvalent character and the semiconducting and diamagnetic properties of a variety of silver oxides and halides. In the particular case of the recently synthesized “idiosyncratic” Ag_7_Pt_2_O_7_ compound,^2^ the relationship between its chemical bonding network and its observed properties poses; however, an extra challenge due to the fact that Ag_7_Pt_2_O_7_ has a number of excess electrons that is not compatible with the number of tetrahedral Ag_4_ voids of the underlying silver sublattice. Jansen and co-workers state that Ag_7_Pt_2_O_7_ has a “composition that violates the basic rules of chemical valence” since only six and not seven silver atoms would be necessary to satisfy the counting of the otherwise electron-precise [Pt_2_O_7_]^6–^ unit.^2^ The odd number of electrons in its unit cell does not help either to understand why this compound shows diamagnetic and nonconducting behavior.

In our previous study of subvalence in the Ag_16_B_4_O_10_ compound, where silver atoms formally display a +0.5 oxidation state,^16^ we briefly introduced an alternative perspective based on the idea of metallic reminiscence. Without resorting to pairwise electron localization, this view was also able to explain the diamagnetic and semiconducting properties of this anomalous borate compound. By metallic reminiscence, we mean that bonding features, interatomic distances, electron density values, and interaction energies of the pure metal element are retained to some extent in some regions of the subvalent compound. Accordingly, the delocalization of the electron density in the parent metallic system decreases in the ionic compound, leading to electron-deficient multicenter bonds only in specific units of the metallic subarray of the compound. As a result, the properties of the parent conventional metal could disappear.

The idea of metallic reminiscence is not new and has been also exploited to rationalize the structures of inorganic crystals^11,17−19^ and electrides,^20−22^ to discuss whether heavy alkali suboxides are electrides, metals, or alkalides,^23^ and also used to detect the most probable sites associated with subvalence in new honeycomb layered Ag_2_M_2_TeO_6_ (M: Ni, Co, Mg, etc.) silver materials.^9,24^ In the latter example, subvalence is not explicitly revealed by the chemical composition but is a result of silver-deficient regions compensated by Ag_6_M_2_TeO_6_ domains containing quasi-independent metallic subvalent silver layers.

The link between metallic reminiscence and subvalence can be especially difficult to confirm in ionic solids such as the Ag_7_Pt_2_O_7_ compound. Assuming +4 and −2 oxidation states for Pt and O atoms, silver atoms with +6/7 oxidation state instead of the formal value of +1 appear as a limiting subvalent case. Topological analysis of scalar fields as the electron density (QTAIM)^25−27^ or the electron localization function (ELF)^28−30^ offers alternative and/or complementary means to discuss experimental results and phenomenological electron counting rules.^31,32^ In spite of some interpretative drawbacks that QTAIM and ELF analysis may introduce when the calculation of electron populations of atoms, bonds, and lone pairs results in noninteger values, these formalisms constitute nonambiguous tools to describe the nature of chemical interactions^33^ since they perform exhaustive and disjoint partitions of the unit cell space of crystalline solids. In particular, QTAIM and ELF have the capability to illustrate how electron delocalization can be spread out across the bulk crystal (metallic bonding),^34−36^ form low dimensional electronic *circuits*,^16^ or collapse in specific regions as tetrahedral Ag units.^1,37^ Moreover, this electron density-based topological analysis can be merged with the evaluation of interaction energies by means of the crystal orbital Hamilton population (COHP) approach,^38^ and with detailed analysis of the electronic band structure to provide a complete and consistent picture from both the wave function and the energy solutions of DFT calculations in the real and reciprocal space.

In this paper, we present a chemical framework aimed at explaining the origin of subvalence in the Ag_7_Pt_2_O_7_ compound. Following a two-step strategy, we first exhaustively examine the particular bonding features of the subjacent metallic Ag_7_Pt_2_ alloy. By means of QTAIM and ELF analysis, we detect silver atoms displaying low oxidation states, how metallic bonding circuits go throughout the alloy, and which atomic regions might present potential subvalent silver clusters. In the second step, we compare these results with those obtained in the Ag_7_Pt_2_O_7_ compound, including the analysis of Ag–Ag interatomic distances, the evaluation of interaction energies, and the identification of the chemical nature of the electronic band structure. Upon oxidation, electron circuits disappear in agreement with the nonconducting behavior of this ionic compound and the electron delocalization collapses just in the silver clusters identified in the analysis performed in the first step. As a result, we not only lay out a procedure to unveil where the electron excess is accommodated in the Ag_7_Pt_2_O_7_ compound but also provide an explanation for the diamagnetic and semiconducting observed properties, associating subvalence with crystalline defects and electrides.

## Computational Details

Unit cell coordinates and lattice parameters for the Ag_7_Pt_2_O_7_ crystal were taken from ref (2). The electronic structure of the oxide compound and its Ag_7_Pt_2_ sublattice were calculated under static conditions within the DFT framework using the VASP code^39^ and the projector augmented wave method.^40^ We used the Perdew–Burke–Ernzerhof (PBE) exchange–correlation functional.^41^ DFT + *U* method was also employed to improve the description of the system based on Dudarev’s formulation.^42^ The values of *U*_eff_ were set to 5.0 eV for Ag^43^ and 7.5 eV for Pt.^44^ The valence configurations 6s^1^5d^9^, 5s^1^4d^10^, and 2s^2^2p^4^ constitute the electronic active space for silver, platinum, and oxygen atoms, respectively. The Brillouin zone was sampled using Γ-centered Monkhorst–Pack meshes,^45^ where the numbers of subdivisions along each reciprocal lattice vector  were given by *N*_*i*_ = int(max(1, 50 + 0.5)). An energy cutoff of 520 eV for the plane waves along with FFT grids of size 144 × 144 × 360 was checked to provide accurate converged energies. The same grids were also used to compute the ELF. Self-consistent iterations were performed until convergence on total energies of 10^–6^ eV was achieved. The electronic band structure at PBE and PBE + *U* levels was calculated in the primitive unit cell. The VASPKIT program^46^ was used to process VASP output, and the *k*-point paths for band calculations were taken from ref (47). The hypothetical Ag_7_Pt_2_S_7_ compound was optimized at the PBE level using the same parameters stated above. 3s^2^3p^4^ valence configuration was used for S atoms. The conjugate gradient algorithm included in the VASP package was employed for the full relaxation of the unit cell.

Spin-polarized HSE06^48^ electronic band structure with symmetry breaking and density of state calculations were carried out using CRYSTAL17 software.^49^ Pob-TZVP-rev2 basis sets^50^ with effective core pseudopotentials were used for Ag and Pt atoms, resulting in a valence space of 19 electrons and 18 electrons, respectively. Pob-TZVP-rev2 all-electron basis sets were used to describe O atoms. Tolerance for the self-consistent field convergence was set to 10^–6^ Hartree. The shrinking factor of the reciprocal space net was set to 12. The different spin states were locked during the first 10 cycles of the electronic minimization procedure, and the latter were allowed to relax. The crystal space group was set to *R*3 ensuring that the most reduced silver atoms, Ag(2), in the primitive cell are nonequivalent. Among the number of different initial magnetic states explored, we notice the choice of assigning either (i) one unpaired electron to one of the silver atoms with a low oxidation state, Ag(2) and Ag(3), or (ii) one electron to the oxygen closer to the subvalent tetrahedral Ag_4_-T silver units.

COHP analysis from VASP wave functions was carried out using the LOBSTER package.^51^ PbeVaspFit2015 atomic basis functions, including the following orbitals for Ag 4d5s, Pt 5d6s, and O 2s2p were used to compute the integrated COHP values. Topological analysis of the electron density and the ELF were carried out using Critic2 code.^52^ Electron density and volume integrations for the different atoms in the unit cell were calculated using the Yu–Trinkle algorithm.^53^ This method recovers the total number of electrons and unit cell volumes with a precision of 99.9%.

### Two Crystalline Fragments and Three Electron-Counting Schemes

#### Platinum and Silver Fragments

**Figure 1:**
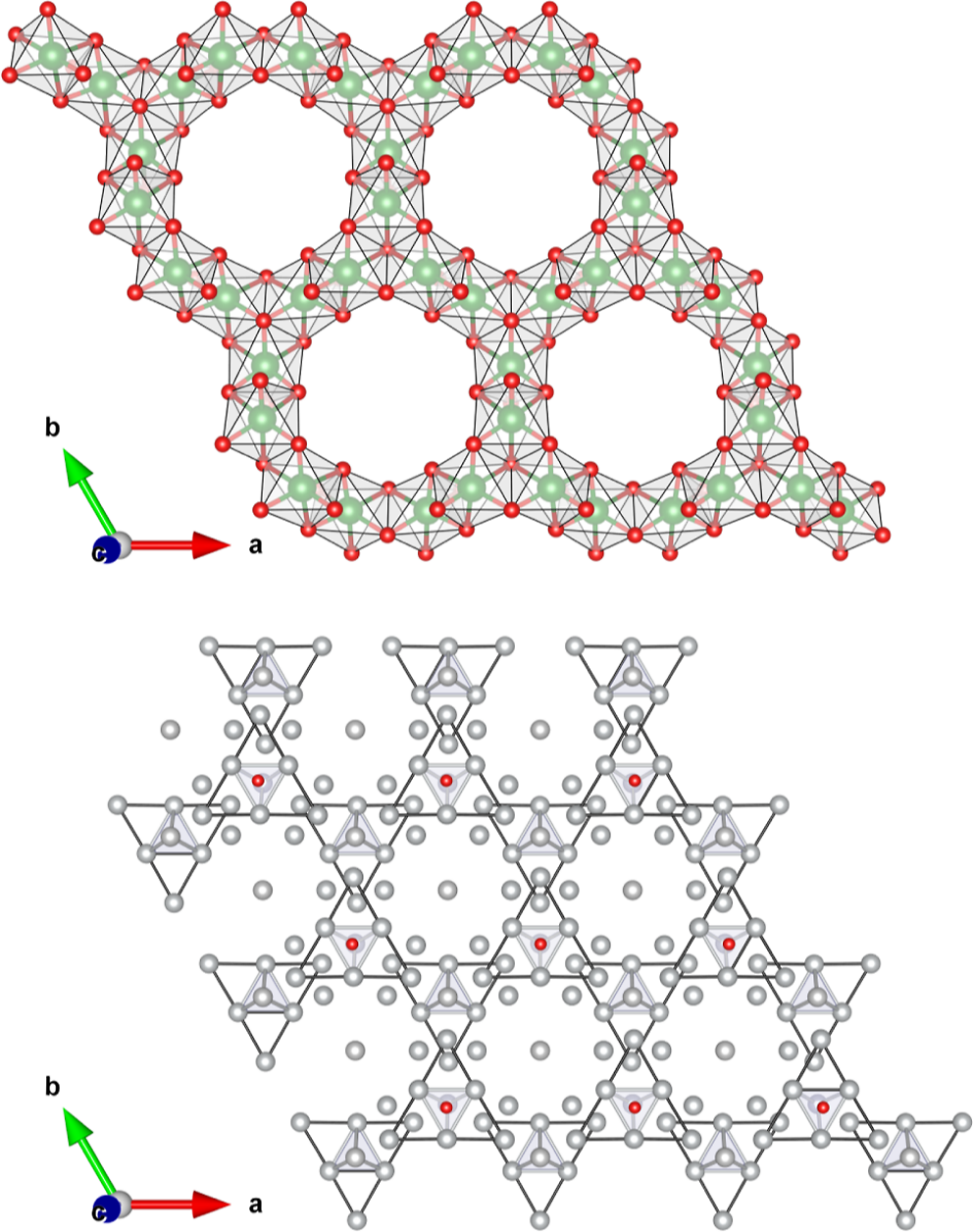
Platinum-oxide (top panel) and silver-oxide (bottom panel) fragments of Ag_7_Pt_2_O_7_. Platinum oxide octahedra and subvalent silver tetrahedra are highlighted. Red, green, and gray spheres stand for oxygen, platinum, and silver atoms, respectively.

To get further insight into the subvalent behavior of this compound without assuming which atoms show nonconventional oxidation numbers, we start by applying an electron counting strategy. An operative procedure allowing the discussion of different possibilities is to carry out a partition of the Ag_7_Pt_2_O_7_ structure splitting it into two fragments with one of the two metallic atoms in each of them (see Figure 1). We pursue an exhaustive and disjoint partition in the sense that the two fragments do not share atoms and complete the whole structure. These two properties are fulfilled by proposing Pt_18_O_60_ (top panel) and Ag_63_O_3_ (bottom panel) compositions for the two fragments. When added, the result is the conventional unit cell with nine Ag_7_Pt_2_O_7_ formula units (*Z* = 9).

The Pt_18_O_60_ fragment consists of slabs of three equivalent layers. Projected along the *c*-axis, the slabs form a honeycomb pattern (see the top panel of Figure 1). In each hexagonal corner, there are three PtO_6_ octahedra connected between them by sharing one edge and forming a Pt_3_O_13_ unit (see Figure S1). If we take into account the number of octahedra that each oxygen is sharing, then we arrive at the [Pt_3_O_10_] building block unit. The three Pt atoms are obviously inside the unit, and among the 13 oxygen atoms, seven are within the unit, and six belong to two [Pt_3_O_13_] units, thus making a total of 10 oxygen atoms.

The remaining Ag_63_O_3_ fragment (see the bottom panel of Figure 1) is also formed by slabs of three silver layers arranged in a honeycomb pattern. Each slab is built by tetrahedral units separated by rhombus-like units. Oxygen atoms in the silver fragment are situated at the Ag octahedral interstices between tetrahedral units, linking two silver slabs as described in detail in ref (2).

#### Electron-Counting Schemes

Three complementary electron counting schemes are possible in this compound depending on whether the oxidation state assumed for Pt atoms is <+4, +4, or >+4, respectively: (i) the electron-excessive, where subvalence occurs both in the platinum and silver fragments, (ii) the electron-precise, with subvalence only in the silver fragment, and (iii) the electron-defective, showing subvalence in the silver fragment and hypervalence in the platinum fragment. The procedure to carry out these electron counting schemes is detailed in the Supporting Information file. Only the electron-specific scheme clearly yields a reasonable result. The possibility of Pt atoms with +2 oxidation states has not been considered since Pt^2+^ usually exhibits square planar environments in opposition to the observed octahedral geometry and would force silver atoms to bear unexpected superoxidation states.

For the defective scheme, a number of possibilities with Pt +4 can be developed, leading to equivalent conclusions. Two illustrative examples are worth to be discussed. In the first one, 18 Ag(0) out of the 63 silver atoms should be found in the unit cell if we want to comply with Wyckoff multiplicities. In this scheme, subvalent silver atoms would be completely identified, yielding a silver fragment with a total of 39 positive charges. This leads to a [Pt_18_O_60_]^39–^ fragment or [Pt_3_O_10_]^6.5–^ in terms of the basic building block. In both options, Pt atoms would hold a fractional oxidation state of +4.5. As each Pt atom should have 18 electrons to obtain a closed shell configuration, the existence of 4c–2e Pt–Pt bonding is mandatory in this counting scheme. (See Supporting Information file for the electron counting procedure). The second defective example involves 12 Ag(0) atoms in the silver fragment. This situation is pertinent since in this case the 12 available electrons would be arranged pairwise in the six tetrahedral interstices of the unit cell along the line suggested by Thakur et al.^2^ Here, the building block would be [Pt_3_O_10_]^7.5–^ with a net charge of +4.17 associated with each platinum atom. Following equivalent reasoning as in the previous example, we again arrive at the condition of Pt–Pt bonding to explain an electron counting consistent with the generalized Lewis’ octet rule. To explain the subvalent character of the idiosyncratic Ag7Pt_2_O_7_ compound, this electron-defective scheme leads again to the conclusion that the concomitant existence of Pt–Pt bonding is mandatory.

Under the excessive electron counting scheme, subvalence is shared between the silver and platinum fragments. Attending to possible multiplicities of the Wyckoff positions, we discuss the prototypical case of 57 Ag^+^ and 6 Ag(0) atoms. Similar to the analysis of the defective scheme, it is easy to show that the total charge of [Pt_3_O_10_] is 8.5-, where the charge of Pt is +3.83 and the total number of electrons associated with Pt exceeds 18. Either the 18-N rule is broken or some of the 2 center Pt–O bonds hold less than 2 electrons.

Each of these two-electron counting schemes involves particular bonding situations. The excessive electron counting does not seem reasonable since the Pt–O distances in Ag_7_Pt_2_O_7_ are very similar to those usually observed in the two center-two electron (2c–2e) bonds of platinum oxide compounds as α-PtO_2_^54^ and PtW_6_O_24_.^55^ Likewise, the defective scheme requires the existence of Pt–Pt bonding. However, neither the topology of the electron density nor the topology of the ELF reveals the existence of these intermetallic bonds in the Ag_7_Pt_2_O_7_ crystal. Moreover, the COHP analyses show a nonbonding interaction between Pt–Pt atom pairs as revealed by close to zero values (COHP_Pt–Pt_ = −0.061 eV/bond). Therefore, we also discarded the electron-defective counting scheme as a potential framework to explain the subvalence in Ag_7_Pt_2_O_7_.

In the electron precise scheme, the building block of the platinum fragment ([Pt_3_O_10_]^−8^) keeps the conventional +4 and −2 oxidation states for Pt and O, respectively. This electron counting scheme leads to a silver fragment with 48 positive charges ([Ag_63_O_3_]^+48^) that would require the existence of nine formally Ag(0) atoms in the unit cell if we assume the common integer (0,+1) oxidation state numbers for silver. According to this electron-precise scheme, subvalence should be shared across the silver fragment since in this unit cell the multiplicities of the silver Wyckoff positions are 18, 6, and 3, but not 9 (Table 1). The question that remains is how, under this scheme, can the nine electrons not transferred to the electron-precise platinum fragment be distributed within the silver fragment. In what follows, we address this question by exhaustively analyzing the peculiar electronic features and structural implications derived from the anomalous oxidation states inherent to the Ag_7_Pt_2_O_7_ stoichiometry.

**Table 1:** Calculated Charges for Ag_7_Pt_2_ Alloy (X = □), Ag_7_Pt_2_O_7_ (X = O), and Ag_7_Pt_2_S_7_ (X = S) along with Their Wyckoff Positions According to PBE + *U* (X = O) and PBE (X = □,S) Calculations^a^

	X = □	X = O			X = S			Wyckoff
	QTAIM	QTAIM	Mulliken	Löwdin	QTAIM	Mulliken	Löwdin	
Pt	–0.32	1.22	1.77	1.48	0.41	0.63	0.62	18h
Ag(1)	0.11	0.51	0.56	0.54	0.35	0.47	0.43	18h
Ag(2)	0.05	0.28	0.15	0.27	0.24	0.22	0.25	6c
Ag(3)	0.09	0.44	0.47	0.50	0.29	0.41	0.40	18g
Ag(4)	0.09	0.44	0.50	0.50	0.26	0.36	0.37	18h
Ag(5)	0.13	0.59	0.62	0.57	0.46	0.65	0.53	3b
X(1)		–0.86	–0.99	–1.00	–0.61	–0.84	–0.84	3a
X(2)		–0.76	–0.94	–0.86	–0.18	–0.28	–0.26	6c
X(3)		–0.80	–0.96	–0.93	–0.38	–0.55	–0.53	18f
X(4)		–0.80	–0.96	–0.90	–0.40	–0.56	–0.53	18h
X(5)		–0.82	–0.97	–0.91	–0.53	–0.72	–0.70	18h

a Values at PBE level for Ag_7_Pt_2_O_7_ are included in Table S1.

### From the Metallic Alloy to the Oxide Compound

Subvalent compounds are characterized by intermetallic distances and structural features akin to those present in the lattices of their metallic constituents. For example, in the borate Ag_16_B_4_O_10_, subvalence was explained by resorting to the residual metallic bonding in the subjacent silver sublattice.^16,37^ Although we noticed in the Introduction that this idea of metallic reminiscence has been previously reported in the literature (see also refs (56 and 57)), in our opinion, it has not been exploited in detail. In this subsection, we carry out a two-step analysis of the electronic structure, starting first with the metallic Ag_7_Pt_2_ sublattice and continuing with the title compound. This strategy is key to identifying electron-deficient multicenter bonding regions in the otherwise ionic bonding network of Ag_7_Pt_2_O_7_.

#### Silver–Platinum Alloy

**Figure 2:**
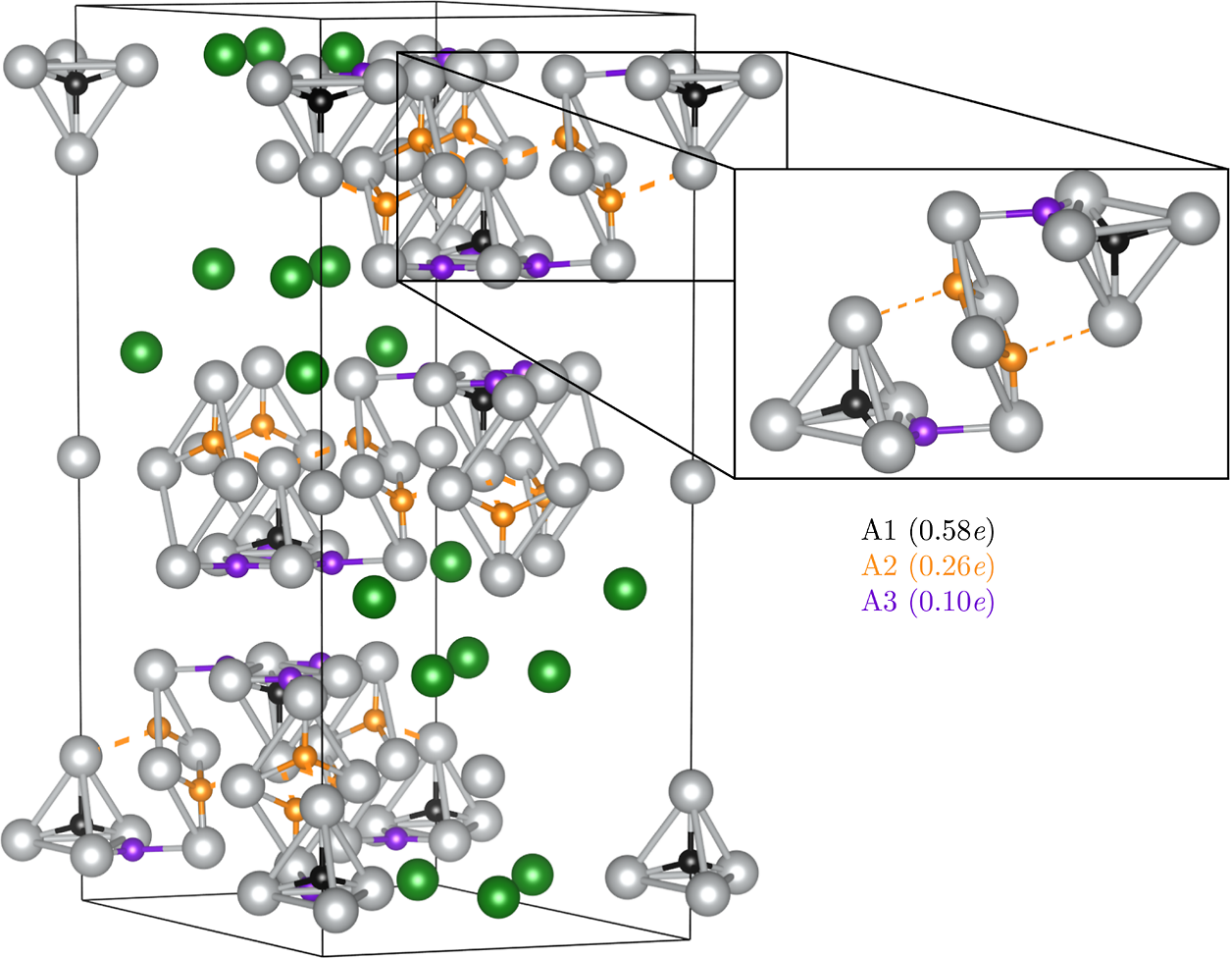
ELF attractors positions in the Ag_7_Pt_2_ metallic sublattice. A1, A2, and A3 attractors are represented as black, orange, and purple spheres, respectively. In the labels, there are the number of electrons between brackets in each attractor. Pt atoms are shown in green and Ag atoms in gray. Zoom with the subvalent silver clusters is provided to facilitate the discussion in the main text.

The subjacent Ag_7_Pt_2_ metallic array of the Ag_7_Pt_2_O_7_ structure shows a slightly distorted face-centered cubic (fcc) packing composed of alternating silver and platinum slabs as displayed in Figure 2. This Ag–Pt alloy is obtained by removing the oxygen atoms from the parent structure at its equilibrium volume. According to our calculations, there is electron transfer from Ag to Pt in the metallic alloy. This charge transference is in agreement with the electronegativity values of Pauling’s scale (χ(Ag) = 1.93, χ(Pt) = 2.28).^58^ Pt atoms hold a charge of −0.32, whereas the degree of oxidation of Ag (from +0.05 to +0.13) increases as its distance is closer to that of Pt atoms. Ag and Pt QTAIM charges are summarized in Table 1.

ELF analysis helps us to picture the metallic bonding features of this alloy. Interestingly, there is a lack of attractors between the Ag–Pt pairs. This result reinforces our view that the structure can be partitioned into independent Ag and Pt subsystems. When focusing on the silver subsystem, we detect three different ELF maxima (attractors, A) located at (i) tetrahedral positions (A1), (ii) rhombus interstices (A2), and (iii) close to the tetrahedra edges (A3) (see Figure 2). ELF values at these positions are all around 0.20. The numbers of electrons in the basins associated with these attractors are 0.58, 0.26, and 0.10 e for A1, A2, and A3, respectively. These values are in the typical range found in the analysis of metallic compounds.^29^ From an orbital perspective, these ELF attractors are associated with the 5s valence electrons of the Ag atoms evidencing silver metallic features in the Ag_7_Pt_2_ alloy. We notice that none of these attractors is situated along the Ag–Ag interatomic line and that the more prominent A1 attractors are located at positions equivalent to the ones found in the fcc structure of pure silver.^16^

**Figure 3:**
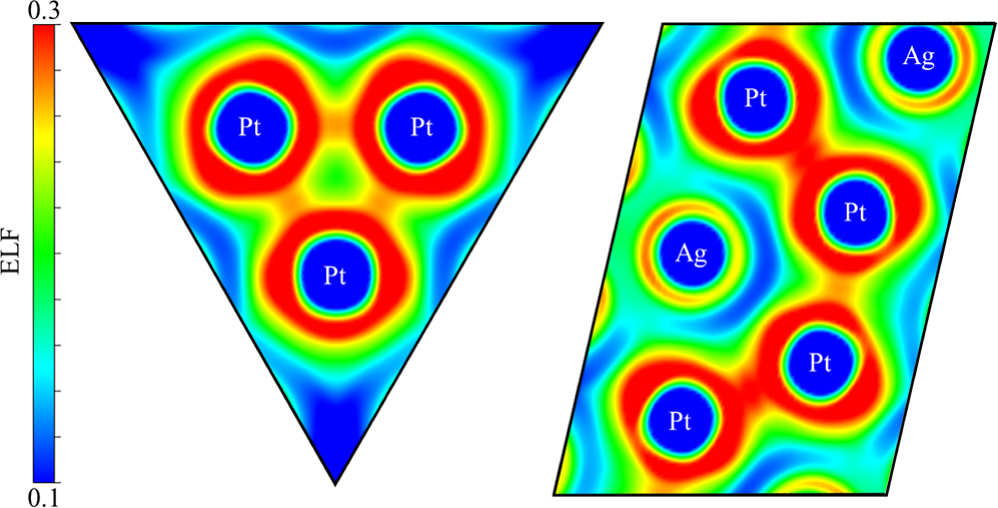
ELF-2D heatmap along [001] and [0 1–1.273] lattice planes showing Pt–Pt bonds in the Ag_7_Pt_2_ metallic matrix.

On the other hand, ELF analysis for the Pt subsystem reveals a directional interaction between Pt atoms (Figure 3). An attractor at the midpoint between each Pt atoms pair is found with an ELF value around 0.30, evidencing a Pt–Pt interaction similar to that found between Pt atoms in dimers and small platinum clusters.^5,7^ The next step is to check to what extent these metallic characteristics are conserved in the Ag_7_Pt_2_O_7_ ionic solid.

#### Silver Platinum Oxide Crystal

When the oxygen constituents of Ag_7_Pt_2_O_7_ are considered in the discussion, two features have to be taken into account: their positions and high electronegativity. Overall, the Pt subsystem is affected both qualitatively and quantitatively when passing from the metallic Ag_7_Pt_2_ alloy to the oxide compound. In terms of QTAIM charges, the values of all equivalent Pt atoms change from −0.32 to +1.22. Since most of the oxygens are directly coordinated to Pt, this notorious effect on the platinum charges is not surprising. Moreover, oxygens clearly modify the ELF topology of the Pt fragment, which does not show Pt–Pt attractors anymore, as we previously anticipated in the analysis of the electron counting schemes. Consequently, the increase in the Pt-oxidation state can be associated with a formal Pt charge of +4 in consonance with an electron-precise [Pt_3_O_10_]^8–^ unit.

The results in the Pt fragment contrast with our findings in the silver subsystem within the Ag_7_Pt_2_O_7_ crystal. As regards QTAIM charges of Ag summarized in Table 1, we observe that the oxidation effect is weaker than in the Pt fragment. A variation of only around 0.5 e in the Ag charge (1.54 e in Pt atoms) is observed when compared with that of the Ag_7_Pt_2_ alloy. Similarly, the ELF analysis reveals that oxygens do not alter so much the topology of the Ag fragment. Although A2 and A3 attractors disappear due to the ionicity increase induced by the oxygen atoms, it is to be highlighted that A1 attractors are still present in the Ag_7_Pt_2_O_7_ compound, but with a smaller number of electrons in the basin (0.12 e).

These results will be discussed in detail in the next section. Here, we can conclude that the different impact of the oxygen atoms in the Pt and Ag fragments is consistent with the electron-precise scheme and the identification of subvalence in the silver fragment and, therefore, constitute a source of relevant information to unveil the origin of the anomalous composition of this Ag_7_Pt_2_O_7_ compound.

### Assessing Subvalence in the Real and Reciprocal Space

The position of the A1 ELF attractors confirms the view proposed in ref (2) since the A1 attractors are precisely located in the same tetrahedral voids that Thakur et al. use to discuss the subvalent character of this compound. In their study, these authors also found an incompatibility between the number of tetrahedral interstices (6) and the number of excess electrons (9) in the unit cell that they circumvent by proposing a supercell structure that “would reconcile all observations made”. However, as admitted by the authors too, the supercell structure “constitutes a particular challenge” that has not yet been conclusively solved yet. Our alternative explanation for this 6 voids-9 electrons problem resorts to chemical arguments related to the metallic reminiscence still present in the compound. Besides the known identification of subvalence in Ag_4_ tetrahedra^1,16^ or Ag_6_ octahedra,^12,13,15^ we have found that the electron excess is also spread throughout other units of the silver subarray. In what follows, we show how subvalence is also manifested in other Ag atoms besides the Ag_4_ tetrahedral units.

**Figure 4:**
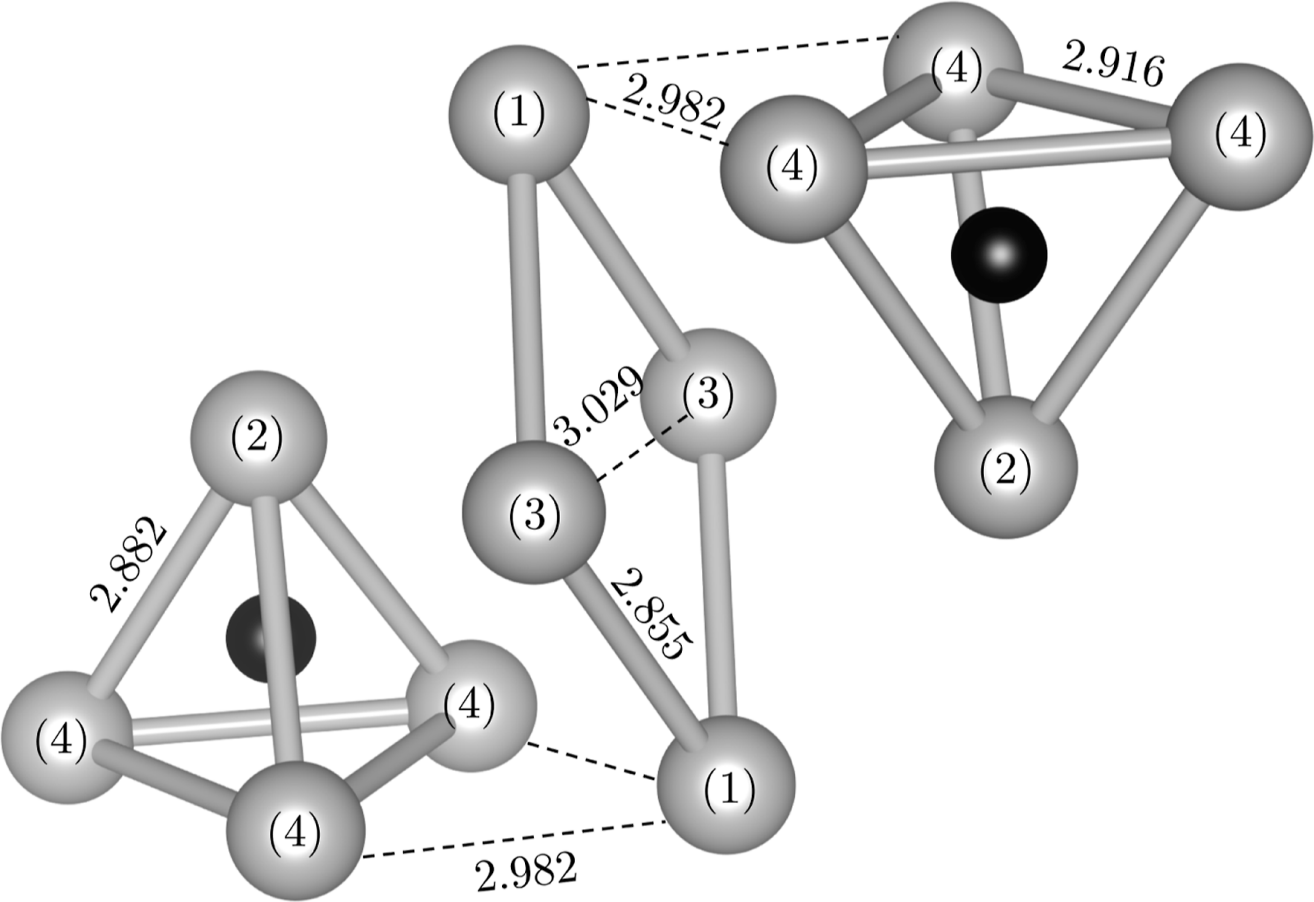
Ag_4_-T and Ag_4_-R motifs of the silver fragment with their corresponding Ag atoms labeled. Ag–Ag distances are given in Å. The number of electrons in the A1 attractor is 0.12 e. ELF attractors are shown as black spheres.

Let us start by analyzing the distances in the Ag_7_Pt_2_O_7_ crystal. Ag–Ag distances similar to or shorter than in the pure metal (2.89 Å) are usually associated with the existence of Ag(0) atoms. This is one reasonable argument that has been used to assign subvalence in this crystal exclusively to Ag_4_ tetrahedra (Ag_4_-T).^2^ We notice however that shorter distances (2.855 Å) are present between silver atoms in the Ag_4_ rhombus motif (Ag_4_-R) located between Ag_4_-T units (see zoom in Figures 2 and 4) suggesting that electron excess could be accommodated in these Ag_4_-R units too. To confirm this expectation, we provide results from several indicators based on both orbital interaction energies and electron density-based topological approaches.

Silver atoms with QTAIM charges smaller than +0.5 are situated in the Ag_4_-T (Ag(2), Ag(4)) and in the Ag_4_-R (Ag(3)) units (see Table 1). Since Ag(3) atoms have the same QTAIM charge as Ag(4) atoms, if we assume that Ag_4_-T units are subvalent, then Ag(3) atoms involved in the Ag_4_-R units should be subvalent as well. Although we are aware that an argumentation only based on charge partitions might not be completely convincing, we also notice that it can be used as a complementary test, and its consistency with the stricter criteria that we discuss below should not be undervalued.

Subvalence has been usually discussed in the playground of orbital overlap and d^10^-d^10^ closed shell interactions.^3,4,6^ A suitable framework to quantify the energies associated with these interactions is the COHP analysis.^38^ It allows a partition of the band structure energy that provides an accurate estimation of the orbital interaction energies between atoms pairs. COHP results in Ag_7_Pt_2_O_7_ are collected in Table 2. The most negative interaction energy (−0.438 eV/bond) is found for the Ag(2)–Ag(4) pair of Ag_4_-T units, which also contains the most reduced silver atom according to QTAIM charges.

**Table 2:** Ag–Ag Distances and COHP Interaction Energies for Different Ag Pairs in the Ag_7_Pt_2_O_7_ Compound According to Our PBE + *U* Calculations^a^

type	*d*_Ag–Ag_ (Å)	*E*_COHP_ (eV/bond)	unit
Ag(2)–Ag(4)	2.882	–0.438	Ag_4_-T
Ag(4)–Ag(4)	2.916	–0.260	Ag_4_-T
Ag(3)–Ag(1)	2.855	–0.324	Ag_4_-R
Ag(3)–Ag(3)	3.029	–0.335	Ag_4_-R
Ag(1)–Ag(4)	2.982	–0.240	R–T

a Values at PBE level are included in Table S2.

From a chemical point of view, the shorter the distance, the lower the interaction energy should be. This is not always the case in the Ag_7_Pt_2_O_7_ compound as values gathered in Table 2 evidence. Our explanation is that the subvalence is responsible for breaking the expected trend.

When comparing distances and interaction energies between Ag(4)–Ag(4), Ag(1)–Ag(3), Ag(3)–Ag(3), and Ag(1)–Ag(4) we observe that in spite of Ag(3)–Ag(3) distances (3.029 Å) being the largest within this subset of silver pairs, its interaction energy is almost the same as the value found for the Ag(1)–Ag(3) pairs, displaying the shortest distance in the crystal. Likewise, the energy of the Ag(3)–Ag(3) interaction is clearly lower than the one of the Ag(4)–Ag(4) (2.916 Å) in the Ag_4_-T unit and also lower than the Ag(1)–Ag(4) (2.982 Å) between Ag_4_-R–Ag_4_-T units.

Being Ag(3)–Ag(3) bonding being stronger and longer than Ag(4)–Ag(4), d^10^-d^10^ dispersive interactions cannot be the only factor responsible for the observed energetic stabilization. A justification for the latter fact is that the electron excess associated with the subvalence of these atoms is employed in stabilizing the Ag(3)–Ag(3) bonds within the Ag_4_-R units through an electron-sharing mechanism. This mechanism would also explain the anomalous distance-energy interaction trend found in this silver compound. We argue that a general link between the subvalence and orbital interaction energies is behind this phenomenon.

**Figure 5:**
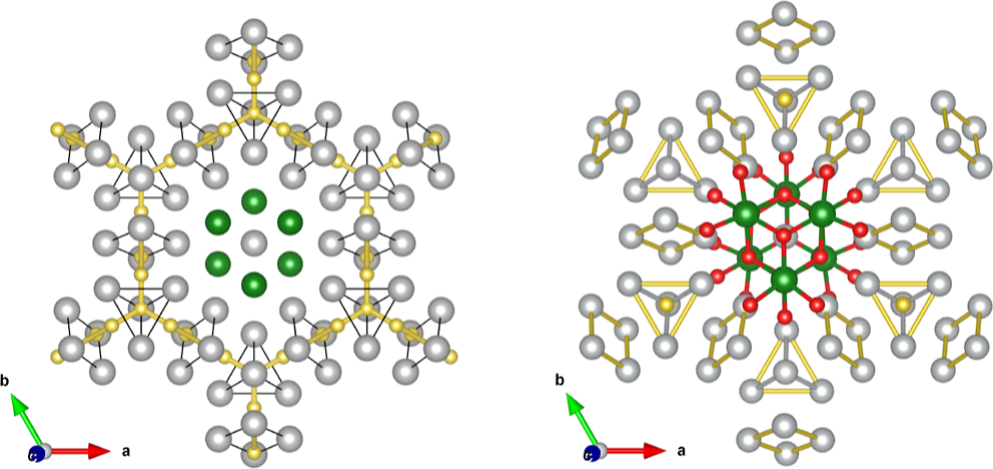
View along the *c*-axis of the ELF attractor circuits (golden color) within one silver slab of the Ag_7_Pt_2_ alloy (top panel) and within the bulk of the Ag_7_Pt_2_O_7_ (bottom panel). Ag_4_-T and Ag_4_-R units are represented by thin yellow lines. Gray, green, and red spheres stand for Ag, Pt, and O atoms, respectively.

Let us conclude our analysis by exploring further the role played by the metallic sublattice in understanding the silver subvalence in the Ag_7_Pt_2_O_7_ compound. The ELF picture of the Ag_7_Pt_2_ alloy detects that both tetrahedral and rhombic units are connected by a basin interconnecting point (bip)^29^ with an ELF value (∼0.165) close to the corresponding values of the attractors (∼0.20). The low difference between the values of ELF at the bip and the attractor point illustrates a delocalization degree similar to the one found in pure fcc silver^16^ and yields ELF circuits as depicted in the top panel of Figure 5. Upon oxidation, the ELF circuit is broken since only the tetrahedral attractors survive, and the connection between the Ag_4_-T and Ag_4_-R clusters is not possible anymore (see Figure 5 bottom panel). Additionally, we note that a bip between silver outercore basins within the rhombic units appears with an ELF value around 0.120, evidencing an electronic delocalization similar but lower than in the tetrahedra.

Our QTAIM, ELF, and COHP descriptors successfully provide an alternative and consistent answer to the 9 electrons and 6 voids problem. First, subvalence is not only located at particular Ag_4_-T units but spread out over other clusters as the Ag_4_-R units. Second, these cluster units present electron-sharing interactions, leading to electron-deficient multicenter bonding. This delocalized multicenter bonding does not involve metallic properties. The insulating Zintl phases with electron-deficient multicenter bonding polyanions^59^ constitute a pertinent example showing a similar bonding pattern. According to our results, the alternative solution for the 9 electron-6 voids problem would be the following: (i) one electron is delocalized in each of the six Ag_4_-T units and (ii) the three electrons left are within the nine Ag_4_-R rhombic units of the unit cell. Therefore, in the Ag_4_-T units, we have a four center one electron (4c–1e) bond, whereas, in each of the Ag_4_-R units, there is a 4c–1/3e bond. We notice that this multicenter bonding view is also compatible with the existence of a superstructure suggested by Thakur et al.^2^ In contrast to the existence of localized electron pairs only in some of Ag_4_ tetrahedra as previously proposed,^2^ our view results in a superstructure with an electronic distribution where all the Ag_4_-T units are similarly occupied and the structure modulation would mainly affect the subvalent nature of the Ag_4_-R units.

To complement the multicenter image of subvalence with the perspective of the reciprocal space, we have also performed a careful analysis of the electronic band structure of Ag_7_Pt_2_O_7_ with varying methodologies including plane wave DFT(PBE) + *U* and more computationally demanding and accurate LCAO calculations at the HSE06 level. Since the unit cell contains an odd number of electrons, we also decided to analyze the effects of spin-polarization in the band structure including symmetry breaking in our LCAO-HSE06 calculations. Consideration of other simulation strategies as supercell calculations to modulate the suggested 2 × 2 × 1 commensurate unit cell^2^ have proved to be computationally prohibitive.

**Figure 6:**
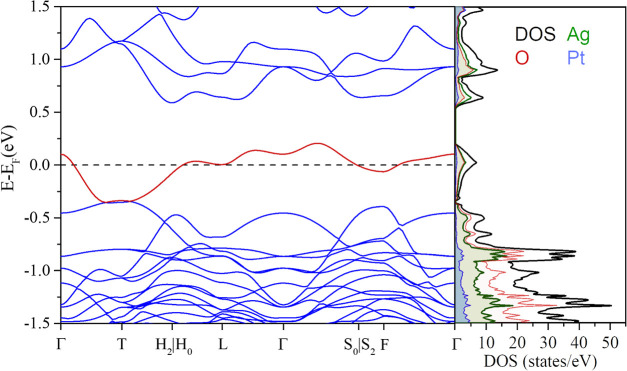
Electronic band structure and the associated atomic and orbital projected density of states of the Ag_7_Pt_2_O_7_ compound at PBE + *U* calculation level. The band at the Fermi level is highlighted in red. The Ag contribution to this band matches the value of the O barely allowing its observation in the PDOS picture.

As one illustrative example from the outputs of this exhaustive exploration, we plot the band structure and corresponding PDOS obtained at the PBE + *U* (*U*(Pt) = 7.5 eV, *U*(Ag) = 5.0 eV) level in Figure 6. Results from PBE (Figure S2) and spin-polarized HSE06 calculations with symmetry breaking (Figure S3) are included in the Supporting Information file. All of these results show a band crossing the Fermi level. Interestingly, the inclusion of correlation effects (PBE + *U* and LCAO-HSE06) or spin-polarization with symmetry breaking at the HSE06 level does not open a conventional band gap. In spite of the number of initial magnetic guesses we have tried, only a negligible difference between the spin-up and spin-down populations (never greater than 0.034) was found (see Figure S3). These results are in agreement with the magnetic measurements performed by Thakur et al.^2^ where the compound exhibits a diamagnetic behavior even at temperatures close to 10 K. According to all these calculations, this picture suggests that although Ag_7_Pt_2_O_7_ might behave as an electric conductor it would not be a conventional metal since the band at the Fermi level barely overlaps with the nearest band at lower energy. More advanced computational methods out of the scope of our chemical analysis of subvalence in Ag_7_Pt_2_O_7_ would be needed to reconcile the reciprocal space picture with the experimental semiconducting band gap of 300 meV determined by temperature-dependent resistivity measurements.^2^

Although all the calculated DFT band structure features of the Ag_7_Pt_2_O_7_ show the incapacity of providing an energy level ordering consistent with a semiconducting picture, the correlated crystalline wave function identifies the existence of a singular band (see band highlighted in red in Figure 6) whose detached nature from the bands above and below is associated with the excess of electrons. This correlated band crossing the Fermi level, evidence that bonding characteristics associated with the subvalent behavior of Ag_7_Pt_2_O_7_ are partially captured, therefore allowing us to complement the conclusions about the subvalence origin we have obtained before in the real space.

**Figure 7:**
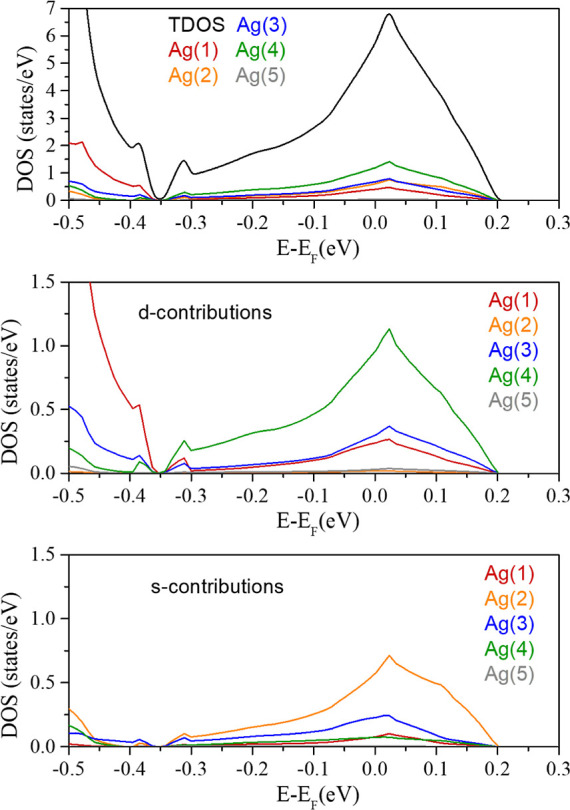
Atomic, d-orbital, and s-orbital contributions of each type of silver atoms to the band crossing at the Fermi level from the projected electronic density of states.

Our PBE + *U* and HSE06 calculations show that this band exhibits Ag 4d–5s hybridization (see Figure 7) with a concomitant oxygen contribution (see PDOS in Figure 6) that has the expected 2p character. In agreement with the multicenter bonding picture associated with the Ag_4_-T and Ag_4_-R units, the orbital projected DOS of the correlated band shows that all of the Ag atoms participate with different weights through their 5s and 4d orbitals (see Figure 7). Besides, the contribution of Ag(2) and Ag(3) 5s orbitals is higher than that of the other Ag atoms, confirming the key role of 5s orbitals in accommodating the electron excess within the silver units. The s and d orbital contributions of the different silver atoms are shown in Figure 7 and data are collected in Table S3. This view is further supported by the analysis of the crystalline orbitals of the band crossing the Fermi level at the occupied *F* and *T* points of the Brillouin zone (see Figure S4). At the *F* point, the orbital nature shows a high participation of the Ag(2) atoms, whereas, in the *T* point, the isosurfaces encompass both the Ag(3) and Ag(2) atoms evidencing that the excess electrons are distributed among the Ag_4_-T and Ag_4_-R units. As a result, the analysis of the electron band structure supports the solution for the 9 electron-6 void problem found in real space.

Under this multicenter bonding view, we can associate subvalence with a crystalline defect in the following manner. The excess of electrons localizes in specific orbitals associated with the Ag_4_-T and Ag_4_-R units, similar to the excess electrons in n-doped crystals^60,61^ or electrons in vacancies of ionic crystals (F-centers).^62^ This electronic localization in structure voids has been theoretically formalized within the interstitial quasi atoms (ISQ) framework proposed by Miao et al.^21,36,63^ Indeed, in a recent study of the high-pressure Na-*hP*4 phase, Racioppi et al. have proposed that the electride nature of this material can be explained proposing a multicenter bonding between the Na atoms forming the structure voids,^64^ similar to the picture we have found in the subvalent Ag_4_-T and Ag_4_-R clusters of the Ag_7_Pt_2_O_7_ compound. We believe that this subvalent “crystalline defect”-ISQ analogy deserves further studies. For instance, the subvalence degree (the number of excess electrons) could be modulated by means of the electronegativity of the nonmetallic atom, since it is responsible for modifying the oxidation state of the silver clusters.

A challenger test to verify the validity of our subvalence vision is to repeat the analysis, replacing oxygen atoms with less electronegative atoms. If excess electrons feel less the attractive action of the nonmetallic atoms, then an increase of the subvalent character of the “same subvalent” silver atoms (as in the oxide compound) would be expected as the multicenter bonding becomes less defective. In other words, the presence of less electronegative nonmetallic atoms should reveal if the metallic reminiscence is a key factor explaining the origin of subvalence.

We carried out this computational experiment in the Ag_7_Pt_2_S_7_ compound using a PBE computational level. PBE charges are similar to the ones calculated using PBE+ *U* as we have demonstrated in the case of Ag_7_Pt_2_O_7_ (see Tables S1 and S4 in the Supporting Information file). QTAIM charges for this compound are collected in Table 1. Compared to the oxide compound, an overall decrease in the oxidation states is observed as expected from the lower electronegativity of sulfur atoms. The same Ag(2), Ag(3), and Ag(4) atoms as in the oxide lattice now show very similar QTAIM charges far from the values of the remaining Ag atoms. Notably, the charges of the Ag(2–4) atoms are now nearly identical to the Ag(2) value involved in the Ag_4_-T unit of the oxide. Two relevant conclusions can be drawn from these results. First, subvalence is again associated with the silver atoms involved in the Ag_4_-T and Ag_4_-R clusters, reinforcing that Ag_7_Pt_2_ metallic characteristics are to some extent retained in the ternary compounds. The second finding is that better identification of subvalent silver atoms is observed.

Finally, let us see how our electronic structure analysis addresses the experimental electric and magnetic observations. Although the electronic band calculations in this odd-electron subvalent compound require further computational efforts beyond standard DFT, they provide a picture of Ag_7_Pt_2_O_7_ as a nonconventional metallic material (see Figure 6). Nevertheless, the semiconducting properties are inferred in the real space where we observe that the 3D and 2D ELF electronic circuits present, respectively, in fcc silver and in the Ag_7_Pt_2_ alloy are broken in the oxide compound. This circuit rupture is evidenced by the existence of electron delocalization regions only within nonconnected isolated cluster units (see Figure 5) and is consistent with the nonmetallic behavior observed in the Ag_7_Pt_2_O_7_ compound. As pure metallic silver is already a diamagnetic material, one would not expect any change in the magnetic behavior in the Ag_7_Pt_2_O_7_ ionic compound.

After considering all of the results discussed in this section, a valid chemical intuitive vision emerges. The role played by the lack of metallic atoms is to disrupt the pure metallic network, generating subvalent clusters within the crystal. As ionicity increases (sulfur to oxygen), an evolution from extended metallic networks to isolated smaller units should be expected. This idea might explain the different degrees of subvalence found in the variety of silver and alkali compounds with anomalous compositions found so far. Besides the ionic interactions, ionic subvalent compounds show electron-deficient multicenter bonding within the isolated clusters resembling Ag(0)–Ag(+1) interactions. In this way, the excess electrons would occupy a band similar to the 5s–5s bonding molecular orbital of the Ag_2_^+^ dimer. We note that this molecule has been observed spectroscopically and determined computationally.^65,66^

## Conclusions

By tracing back the positions where delocalized electrons are in the subjacent metallic Ag_7_Pt_2_ alloy, we were able to foresee the atomic clusters with electron-deficient multicenter bonding in the Ag_7_Pt_2_O_7_ oxide. This strategy can be applicable to other subvalent compounds. Since these bonding patterns are enhanced when oxygen is replaced by sulfur in a hypothetical Ag_7_Pt_2_S_7_ crystal, a correlation between the electronegativity of the nonmetallic atom and the degree of subvalence of the compound is suggested. The lower the electronegativity, the higher the number of electrons shared across the subvalent units. This rule can be of utility to anticipate or guide the proposal of new subvalent compounds.

A new chemical perspective of subvalence is provided introducing electron delocalization (multicenter bonding) within silver units where metallic reminiscence was identified. In addition to the previously proposed Ag_4_-T tetrahedra, Ag_4_-R rhombus-shaped clusters displaying the shortest Ag–Ag distances in the compound have been unveiled with silver atoms with low oxidation state silver atoms. These Ag_4_-T and Ag_4_-R clusters form isolated regions in the unit cell with 4c–1e and 4c–1/3e bonds, respectively, preventing bulk electron conduction in the compound and providing an alternative solution to the 9 electrons-6 voids problem. Magnetic behavior is not expected either, since pure silver is diamagnetic and the presence of subvalence in isolated regions of the otherwise Ag_7_Pt_2_O_7_ ionic compound does not add any meaningful paramagnetic contribution. The electron excess in these two types of clusters is associated with particularly low Ag–Ag interaction energies resembling bonding states of small metallic Ag clusters.^67,68^ Besides, we propose an analogy between subvalence and crystalline defect-ISQ that might be used to guide the engineering of subvalent and electride compounds. We believe that these ideas could be of general application to other subvalent compounds and are worth further exploring.

## Supporting Information Available

Additional computational data and electron counting schemes are provided (PDF)
