# Supplementary material for: Where are the Excess Electrons in Subvalent Compounds? The Case of Ag7Pt2O7
Source: Inorg Chem. 2024 Mar 18;63(13):5897–907. doi: 10.1021/acs.inorgchem.3c04409 (PMC10988551; doi:10.1021/acs.inorgchem.3c04409)
Supplement: Supplementary file 1 — ic3c04409_si_001.pdf [file ic3c04409_si_001.pdf]

Where are the excess electrons in subvalent compounds? The case of $Ag_7Pt_2O_7$

Fernando Izquierdo-Ruiz,[†] Miguel Angel Salvadó,[‡] Alvaro Lobato,[*,†] and Jose Manuel Recio[‡]

[†]MALTA-Consolider Team and Departamento de Química Física, Universidad Complutense de Madrid. E-28040 Madrid, Spain

[‡]MALTA-Consolider Team and Departamento de Química Física y Analítica, Universidad de Oviedo. E-33006 Oviedo, Spain

* Corresponding author: a.lobato@ucm.es

Table of Contents

Results.. **2**

Electron Counting Schemes.. 2

Electronic structure calculations.. 3

References.. **4**

Author Contributions.. **4**

Results

Electron Counting Schemes:

We start with the electron-precise counting scheme with Pt +4 ions. Our partition leads to a $[Pt_{18}O_{60}]$ fragment holding 48 negative charges, since a -2 charge is considered for all oxygen atoms. In this scheme the $[Pt_3O_{10}]^{8-}$ building block can be written as $[Pt_2O_{20/3}]^{16/3-}$. Incidentally, this is in contrast with the unit proposed in Ref. [1], where all the oxygen atoms are considered in a $Pt_{18}O_{63}$ sublattice yielding to $[Pt_2O_7]^{6-}$ units in this scheme. All the sub-valence resides in the $[Ag_{63}O_3]^{48+}$ unit where 9 formally Ag(0) atoms would be found if we assume the common integer (0, +1) oxidation state numbers for the silver atoms. According to this electron-precise scheme, silver sub-valence should be shared across the silver fragment since in this unit cell the multiplicities of silver Wyckoff positions are 18, 6, and 3, but no 9.

For the defective scheme, a number of possibilities with Pt >+4 can be developed leading to equivalent conclusions. Two illustrative examples are worth to be discussed. In the first one, 18 Ag(0) out of the 63 silver atoms should be found in the unit cell if we want to comply with Wyckoff multiplicities. In this scheme, subvalent silver atoms would be completely identified yielding a silver fragment with a total of 39 positive charges. This leads to a $[Pt_{18}O_{60}]^{39-}$ fragment or $[Pt_3O_{10}]^{6.5-}$ in terms of the basic building block. In both options, Pt atoms would hold a fractional oxidation state of +4.5.

Using the simple $[Pt_3O_{10}]$ unit, we can anticipate the bonding of this first example of the defective electron counting scheme by means of a Lewis structure analysis. In Figure S1, we see that there are 3 apical oxygen atoms singly coordinated to a Pt atom, 6 oxygen atoms coordinated to two Pt atoms, and 1 oxygen coordinated to 3 Pt atoms. Monocoordinated, bicoordinated and tricoordinated oxygen atoms hold 3, 2, and 1 lone electron pairs, respectively. This yields a total of 3·3+6·2+1·1 = 22 oxygen lone electron pairs. As the total number of valence electrons in the $[Pt_3O_{10}]^{6.5-}$ is 96.5*e* (3·10+10·6+6.5) and the total number of electrons to complete each atom shell, assuming the 18-electron rule for the Pt atoms, is 134*e* (3·18+10·8), the number of bonding electrons is 37.5 (134-96.5).

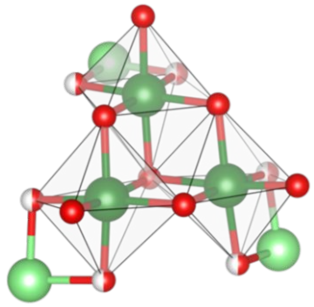

Figure S1. Scheme of the Pt_3O_{10} building block within the Pt_6O_{13} unit. Notice that shared oxygens are half-colored in red and white and platinum atoms not belonging to the building block are in light green.

Let us now get the number of electrons in the Pt atoms. Subtracting the number of oxygen lone pair electrons to the total number of valence electrons (96.5-22·2) in the $[Pt_3O_{10}]^{6.5-}$ unit, we obtain that each Pt atom bears 17.5*e*. As each Pt atom should have 18 electrons to obtain a closed shell configuration, the existence of 4c-2e Pt-Pt bonding is mandatory in this counting scheme where 18 Ag(0) subvalent atoms are identified.

The second defective example involves 12 Ag(0) atoms in the silver fragment. This situation is pertinent since in this case the 12 available electrons would be arranged pairwise in the six tetrahedral interstices of the unit cell along the line suggested by Thakur *et al.*[8] Here, the building block would be $[Pt_3O_{10}]^{7.5-}$ with a net charge of +4.17 associated to each platinum atom. Following equivalent reasoning as in the previous example, we again arrive at the condition of Pt-Pt bonding to explain an electron counting consistent with the generalized Lewis' octet rule. To explain the sub-valent character of the idiosyncratic $Ag_7Pt_2O_7$ compound, this electron-defective scheme leads to the conclusion that the concomitant existence of Pt-Pt bonding is mandatory. The third and last counting scheme involves a number of Ag(0) atoms lower than in the electron-precise situation. Under this scheme, sub-valence is shared between the silver and platinum fragments. Attending to possible multiplicities of the Wyckoff positions, we discuss the prototypical case of 57 Ag^+ and 6 Ag(0) atoms. Similar to the analysis of the defective scheme, it is easy to show that the total charge of $[Pt_3O_{10}]$ is 8.5-, where the

charge of Pt is +3.83 and the total number of electrons associated with Pt exceeds 18. Either the 18-N rule is broken or some of the 2 centers Pt-O bonds hold less than 2 electrons.

Electronic Structure Calculations

Table S1. QTAIM charges (q) in the $Ag_7Pt_2O_7$ compound at PBE and PBE+U levels.

	Wyckoff	q_{PBE}			q_{PBE+U}
Pt	18h	1.37	1.61	1.46	1.22
Ag(1)	18h	0.54	0.57	0.54	0.51
Ag(2)	6c	0.27	0.16	0.28	0.28
Ag(3)	18g	0.46	0.48	0.50	0.44
Ag(4)	18h	0.46	0.50	0.51	0.44
Ag(5)	3b	0.63	0.64	0.57	0.59
O(1)	3a	-0.92	-1.01	-1.02	-0.86
O(2)	6c	-0.84	-0.92	-0.86	-0.76
O(3)	18f	-0.86	-0.94	-0.90	-0.80
O(4)	18h	-0.86	-0.95	-0.90	-0.80
O(5)	18h	-0.87	-0.95	-0.93	-0.82

Table S2. COHP data in $Ag_7Pt_2O_7$ at PBE and PBE+U levels. Labels are the same as those presented in the main text.

Type	$d_{Ag\text{-}Ag}$ (Å)	$E_{COHP\text{-}PBE}$ (eV/bond)	$E_{COHP\text{-}PBE+U}$ (eV/bond)	Unit
Ag(2)-Ag(4)	2.882	-0.462	-0.438	Ag_4-T
Ag(4)-Ag(4)	2.916	-0.287	-0.260	Ag_4-T
Ag(3)-Ag(1)	2.855	-0.378	-0.324	Ag_4-R
Ag(3)-Ag(3)	3.029	-0.368	-0.335	Ag_4-R
Ag(1)-Ag(4)	2.982	-0.276	-0.240	R-T

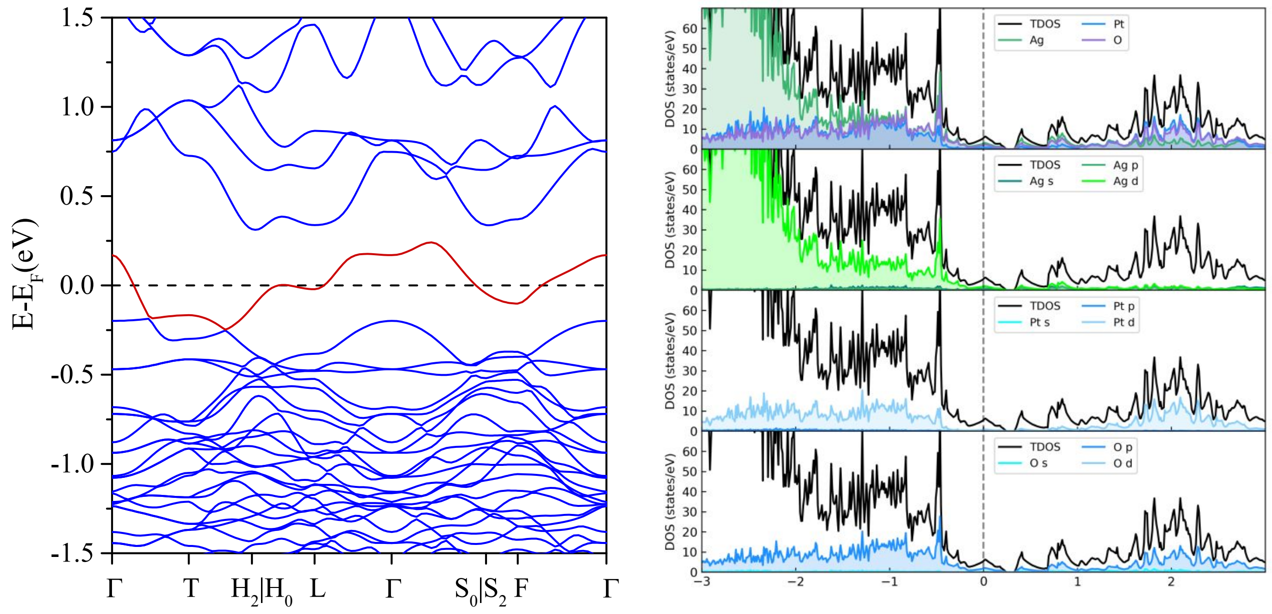

Figure S2. Electronic band structure and the associated atomic and orbital projected density of states of the $Ag_7Pt_2O_7$ compound at PBE level of calculation.

Regardless the magnetic guesses we tried, spin polarized calculations at LCAO-HSE06 level with symmetry breaking result in a negligible difference between spin up and spin down populations, with values never greater than 0.034. This effect is appreciated in Fig. S3: the dashed and solid lines perfectly overlap except at the Fermi level, where the difference is hardly noticeable. In this case, we have employed a different but equivalent *k*-path for the electronic band structure calculations as recommended by *seek-path* tool.[10] Notice that at this level of calculation, the intermediate band is well separated from the valence-like and conduction-like bands.

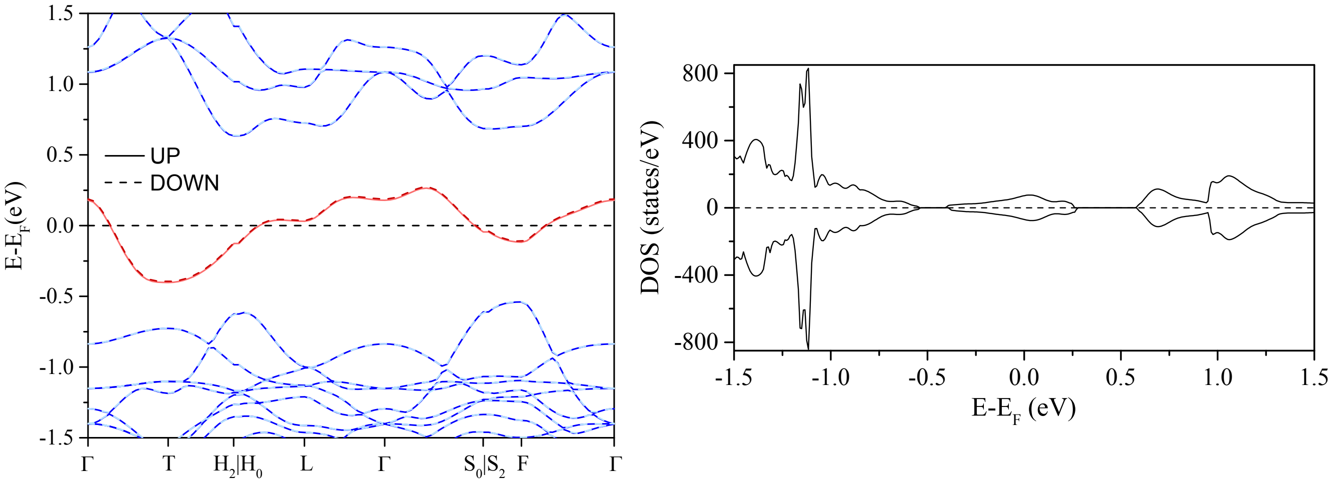

Figure S3. Spin polarized electronic band structure and the associated density of states calculated at LCAO-HSE06 level. Spin up and spin down bands are plotted with solid and dashed lines, respectively. Up and down bands at the Fermi level are shown in red.

Table S3. Number of electrons in *s, p* and *d* orbitals and in each type of Ag atoms obtained from the DOS integration of the band crossing the Fermi level in the primitive unit cell. Values are weighted according to the multiplicities of each silver atom.

	Multiplicity	*s*	*p*	*d*	Total
Ag(1)	6	0.024	0.024	0.060	0.108
Ag(2)	2	0.148	0.004	0.004	0.156
Ag(3)	6	0.072	0.048	0.072	0.192
Ag(4)	6	0.024	0.036	0.288	0.348
Ag(5)	1	0.004	0.002	0.008	0.014

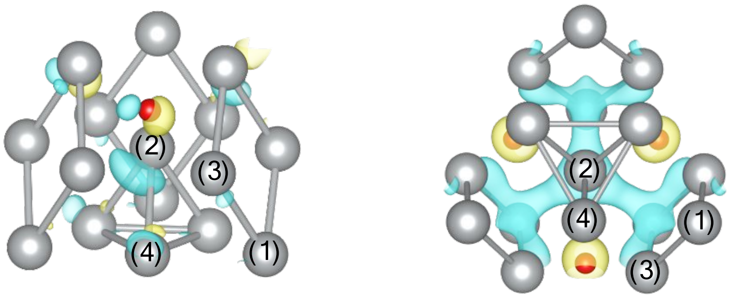

Figure S4. Isosurfaces of the real part of crystalline orbitals associated with the intermediate band at the occupied F (left panel) and T(right panel) points of the Brillouin zone. The same isovalue has been used to illustrate the contribution of the different Ag atoms to the crystalline orbital. For the sake of clarity, we show only the fraction of the unit cell related with the Ag_4-T and Ag_4-R units.

Table S4. Number of electrons (N_{A1}) and ELF value (η_{A1}) of the A1 attractor and ELF at the bip (η_{bip}) between silver outercore basins within the rhombic units in $Ag_7Pt_2O_7$ at PBE and PBE+U levels.

	PBE	PBE+U
N_{A1}	0.27	0.12
η_{A1}	0.185	0.163
η_{bip}	0.155	0.120

References

[1] Thakur, G. S.; Dinnebier, R.; Hansen, T. C.; Assenmacher, W.; Felser, C.; Jansen, M. Idiosyncratic $Ag_7Pt_2O_7$: An Electron Imprecise yet Diamagnetic Small Band Gap Oxide. *Angew. Chem. Int. Ed.*, **2020**, 59, 19910.

Author Contributions

J.M.R. and A.L. designed research; A.L., F.I., and M.A.S. performed the calculations; J.M.R., A.L., F.I., and M.A.S. wrote the original draft of the paper; J.M.R., A.L., F.I., and M.A.S. revised and edited the paper.
